# The accuracy and reliability of digital measurements of gingival recession versus conventional methods

**DOI:** 10.1186/s12903-019-0851-0

**Published:** 2019-07-16

**Authors:** Hytham N. Fageeh, Abdullah A. Meshni, Hassan A. Jamal, Reghunathan S. Preethanath, Esam Helboub

**Affiliations:** Division of Periodontics, College of Dentistry, Jazan University, P.O.Box 114, Jazan, 45142 Saudi Arabia

**Keywords:** Gingival recession, Intraoral scanning, Cast model, Intra-class coefficient

## Abstract

**Background:**

An apical shift in the position of the gingiva beyond the cemento-enamel junction leads to gingival recession. This study aimed to evaluate the reproducibility of digital measurements of gingival recession when compared to conventional measurements taken clinically using periodontal probes.

**Methods:**

Gingival recession was measured at 97 sites in the oral cavity by four examiners using the following methods: CP, direct measurement of gingival recession using William’s periodontal probe intraorally; CC, measurements on cast models using a caliper; DP, digital measurement on virtual models obtained by intraoral scanning, and DC, digital measurements on virtual models of dental casts. Intra-class and inter-rater correlations were analyzed. Bland Altman plots were drawn to visually determine the magnitude of differences in any given pair-wise measurements.

**Results:**

In this study, good inter-methods reliability was observed for almost all the examiners ranging from 0.907 to 0.918, except for one examiner (0.837). The greatest disagreements between the raters were observed for methods; CP (0.631) followed by CC (0.85), while the best agreements were observed for methods DP (0.9) followed by DC (0.872).

**Conclusion:**

Variations in measurements between examiners can be reduced by using digital technologies when compared to conventional methods. Improved reproducibility of measurements obtained via intraoral scanning will increase the validity and reliability of future studies that compare different treatment modalities for root coverage.

**Electronic supplementary material:**

The online version of this article (10.1186/s12903-019-0851-0) contains supplementary material, which is available to authorized users.

## Background

Gingival recession refers to the exposure of the surface of the root following an apical shift in the position of the gingiva beyond the cemento-enamel junction (CEJ) [1, 2]. It is generally seen in adults, and may be localized or generalized, involving one of more teeth. The gingiva comprises epithelial and connective tissues and forms a collar around the neck of the tooth [3]. The parts of the gingival epithelium include the oral gingival epithelium, the sulcular gingival epithelium, which lines the gingival sulcus, and the junctional epithelium, which attaches the gingiva to the tooth [3, 4].

Gingival recession is caused by several factors such as anatomical abnormalities (thin alveolar bone or gingival tissue, deficient keratinized mucosa, tooth malposition, high frenal attachment), trauma (tooth brushing), inflammation (due to presence of plaque or calculus) and from iatrogenic factors such as improper denture design, placement of orthodontic appliances or restorations [1, 5, 6]. In a healthy periodontium, the gingiva is positioned 0.5 to 2.0 mm coronal to the CEJ, and a shift from its normal position beyond the CEJ results in gingival recession [7]. Clinically, gingival recession is measured in millimeters from the gingival crest to the CEJ, using a dental probe; however, this method is thought to be semi-quantitative and inaccurate [8]. Plaster models may prove useful in cases where it is difficult to measure recession intraorally, as they provide a three-dimensional (3D) view allowing for detailed assessments of the impressions obtained during clinical examination without interference from soft tissues within the confines of the oral cavity [9]. However, the disadvantages of study casts include, physical and chemical damage, wear and tear, and distortion [10]. In addition, the use of plaster models is neither time- nor cost-effective. Thus, digital models were introduced in the late 1990s. The advantages of using digital models include ease of handling and storage, time-effectiveness, and reduced manual errors since data can be electronically transferred and stored. Digital models may be obtained via scanning of the intraoral tissues (creating virtual models) or study casts (creating digital cast models). Intraoral scanners are devices used for capturing direct optical impressions in dentistry [11, 12]. The dental arches are scanned, images of the oral tissues are captured and processed, and a 3D virtual model is finally created [12]. Similarly, plaster models are scanned using 3D scanners to create digital images. These advances in technology have proved extremely useful as diagnostic tools in dentistry.

In the present study, we aimed to investigate the reproducibility and reliability of digital measurements of gingival recession when compared with the conventional methods (dental probe, study casts).

## Methods

This study was performed at the College of Dentistry, Jazan University, from September 2017 to February 2018. Fifteen volunteers exhibiting a gingival recession of at least 2 mm were enrolled in this study. The participants aged between 20 and 50 years were screened clinically to exclude those with systemic illnesses. The following exclusion criteria were used: use of any type of medication over the past two or more weeks; presence of any chronic medical condition, including diabetes or viral, fungal or bacterial infections; history of physical trauma during the previous 2 weeks; presence of aggressive periodontitis, periodontal abscess, or necrotizing ulcerative gingivitis/ periodontitis; any periodontal treatment and/or antibiotic therapy received during the preceding 3 months; any type of dental work or tooth extraction(s) performed over the last 2 weeks; and refusal to sign the consent form.

Ethical approval was obtained by the ethical committee of the scientific research unit, College of Dentistry, Jazan University under the reference number: CODJU-1709I and a written informed consent was obtained from all participants.

Gingival recession was measured as the distance from the CEJ to the gingival margin (GM), parallel to the long axis of the tooth starting from the most apical point of recession. The height of the mesial papilla was measured from a line connecting the cusp tips or incisal edges of the adjacent teeth to the tip of the papilla parallel to the long axis of the tooth. The mesial papilla was chosen due to better visual accessibility. Consistency in measurements between recession and papilla height was obtained by this method.

A total of 97 sites were evaluated via direct clinical and digital measurements.

**Fig. 1:**
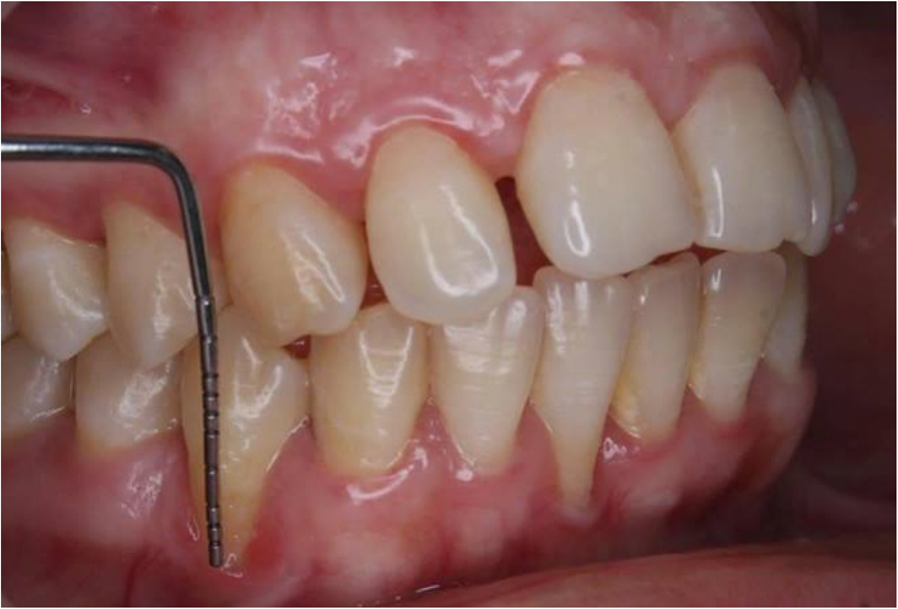
Direct measurement of gingival recession using a William’s periodontal probe (CP)

**Fig. 2:**
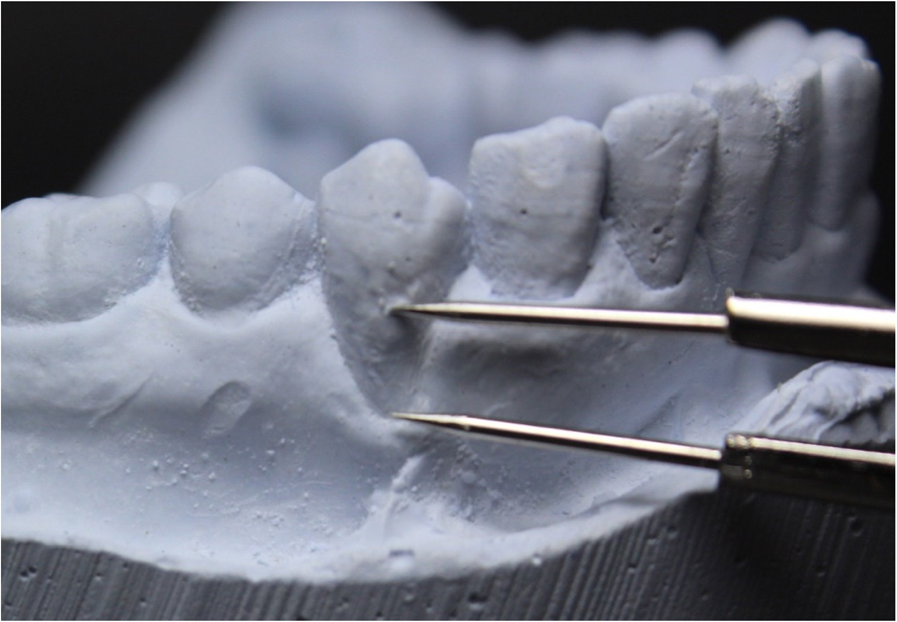
Measurement of gingival recession on cast models using a caliper (CC)

The two conventional methods used for the direct clinical measurements were as follows:

- Measurements in the oral cavity using a calibrated William’s periodontal probe (CP) (Fig. 1)
- Measurements on cast models using a caliper (CC) (Fig. 2). Polyether impressions were taken using customized impression trays, and cast models were fabricated. A caliper with a 10-mm scale was used for the linear measurements.

**Fig. 3:**
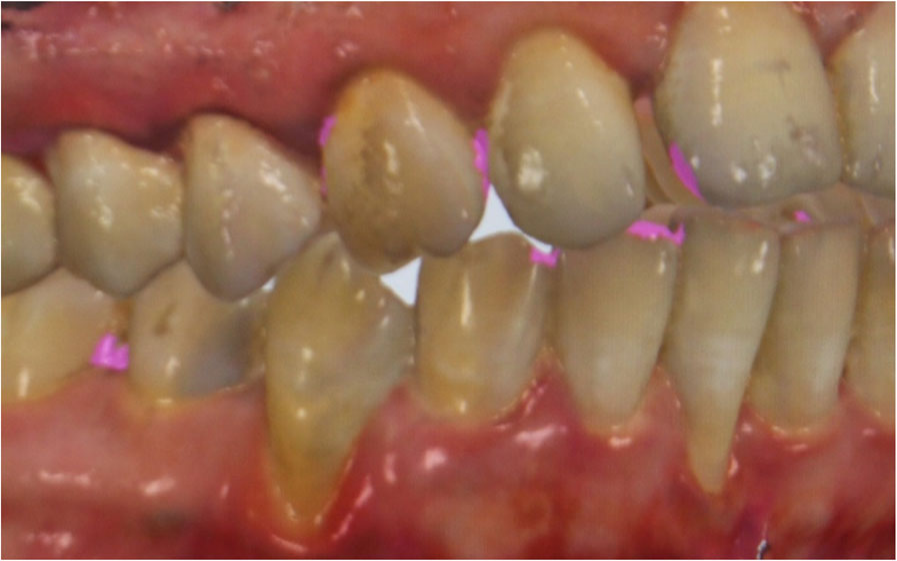
Measurements on virtual models obtained from intraoral optical impressions using 3 shape software program (DP)

**Fig. 4:**
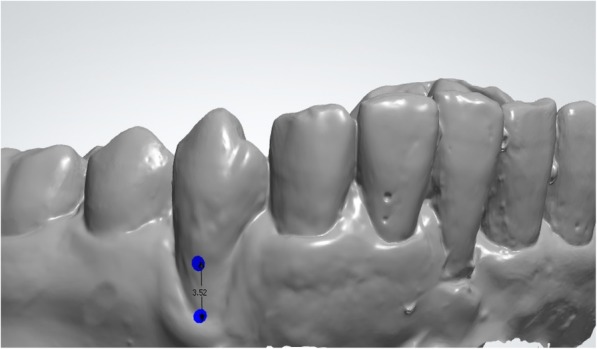
Measurements on virtual models obtained from optical impressions of cast models using the 3 shape software (DC)

Digital measurements were obtained using the following two methods:

- Measurements on virtual models obtained from intraoral optical impressions using Trios 3 shape software program (DP) (Fig. 3).
- Measurements on virtual models obtained from optical impressions of cast models using Trios 3 shape software (DC) (Fig. 4).

All measurements were performed by four examiners (3 faculty members and 1 intern at the school where the study was conducted) in random order using a computer-generated randomization list. The obtained data from the measurements were entered into a data extraction table, which was not accessible to the examiners.

### Statistical analysis

Statistical analysis of the data was performed using SPSS Version 22 (Armonk, New York: IBMCorp.) and MedCalc for Windows, version 15.0 (MedCalc Software, Ostend, Belgium). The means, standard deviations (SD), and standard errors (SE) of the measured recessions were presented. Intra-class correlation coefficients (ICC), along with their 95% confidence intervals (CI) were used to evaluate the inter-method and inter-examiner reliabilities of gingival recession measurements obtained from the 97 sites in the oral cavity. Based on the study by Landis and Koch (1997), the ICC scale was interpreted as follows: poor to fair (below 0.4), moderate (0.41–0.60), excellent (0.61–0.80), and almost perfect (0.81–1) [13].

In order to depict the pair-wise variations between each pair of methods/examiners, Bland and Altman Plots were drawn displaying the mean values for each pair against the difference, and demonstrating the degree of agreement between the examiners or methods. The difference for each point, the mean difference, and the confidence limits are illustrated on the vertical axis, while the average of two measurements are depicted along the horizontal axis [14]. Of the four horizontal lines in the graph, the middle blue line represents the observed difference in mean values, and dotted red line in the middle indicates the expected mean difference (zero). The two lines on the top and bottom indicate the 95% confidence limits within which about 95% of the differences between the measurements of each examiner or method should lie [15]. The within-examiners and within-methods biases along with their 95% upper and lower limits were calculated.

## Results

Table 1 shows the mean and SD values of gingival recession measured by the four examiners using the four different methods. The highest mean was reported by examiner B using the conventional probe model (CP; 2.24 ± 0.97 mm), while the lowest was reported by examiner D using DC (1.64 ± 0.74 mm). The method with the highest mean value obtained by combining the measurements taken by all four examiners was CP (2.11 ± 1 mm), and the one with lowest mean value was CC (1.91 ± 0.7 mm). The highest score for all methods combined was measured by examiner B (2.16 ± 0.85 mm), and the lowest by examiner D (1.79 ± 0.8 mm).

**Table 1:** Means, standard deviations (SD) and standard errors (SE) of the recession measurements by individual examiners and methods, and for all methods and all examiners combined

Examiner	Method	Recession Measurement^a^		
		Mean	SD	SE
A	CP	2.23	1.38	0.14
	CC	1.95	0.84	0.09
	DP	2.19	0.8	0.08
	DC	2.16	0.79	0.08
B	CP	2.24	0.97	0.1
	CC	2.06	0.86	0.09
	DP	2.17	0.80	0.08
	DC	2.17	0.77	0.08
C	CP	2.14	1.12	0.11
	CC	1.98	0.97	0.1
	DP	2.16	0.85	0.08
	DC	2.12	0.82	0.08
D	CP	1.91	0.94	0.1
	CC	1.64	0.74	0.07
	DP	1.85	0.83	0.08
	DC	1.77	0.65	0.07
CP all (*N* = 392)		2.11	1	0.05
CC all (*N* = 392)		1.91	0.87	0.04
DP all (*N* = 392)		2.09	0.83	0.04
DC all (N = 392)		2.06	0.78	0.04
A all (*N* = 392)		2.11	0.85	0.04
B all (*N* = 392)		2.16	0.85	0.04
C all (*N* = 392)		2.1	0.95	0.05
D all (*N* = 392)		1.79	0.8	0.04

^a^: *N* = 98 unless stated otherwise. A, B, C, and D: the four examiners in the study. CP, conventional method using periodontal probe; CC, conventional method of taking measurements on cast model using caliper; DP, digital measurements of intraoral scans; and DC, digital measurements of digitized cast models

Table 2 shows the values of the intra-class correlation coefficients (ICCs) for the inter-method, inter-examiner, all methods, and all examiners. The ICCs for all methods combined, irrespective of the examiners, and for all examiners combined, irrespective of the methods, were almost perfect 0.933 and 0.912, respectively.

**Table 2:** Intraclass correlation coefficients (ICC) for inter-methods, inter-examiners, and all methods and all examiners combined

Examiner	Method	ICC	95% ICC
A	CP	0.911	0.869–0.940
	CC		
	DP		
	DC		
B	CP	0.907	0.867–0.936
	CC		
	DP		
	DC		
C	CP	0.918	0.886–0.943
	CC		
	DP		
	DC		
D	CP	0.837	0.759–0.891
	CC		
	DP		
	DC		
Method	Examiner	ICC	95% ICC
CP	A	0.631	0.495–0.737
	B		
	C		
	D		
CC	A	0.850	0.765–0.903
	B		
	C		
	D		
DP	A	0.900	0.849–0.933
	B		
	C		
	D		
DC	A	0.872	0.788–0.920
	B		
	C		
	D		
Agreement between		ICC	95% CI
All Methods		0.933	0.920–0.944
All examiners		0.912	0.887–0.931

A, B, C, and D: the four examiners in the study. CP, conventional method using periodontal probe; CC, conventional method of taking measurements on cast model using caliper; DP, digital measurements of intraoral scans; and DC, digital measurements of digitized cast models

**Fig. 5:**
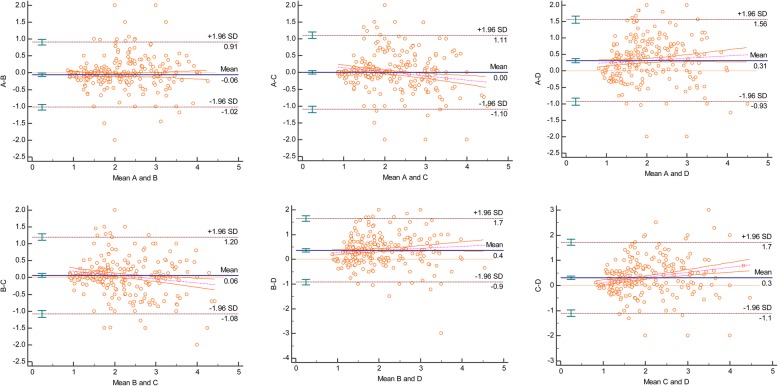
Bland and Altman analysis showing the extent of agreement among the four examiners (A, B, C, D)

Differences between examiners, irrespective of the methods used, are illustrated in the Bland and Altman plots in Fig. 5 and presented in Table 3. The least differences were found between examiners A and C (− 0.004 mm), A and B (0.055 mm), followed by B and C (− 0.056 mm). In spite of the low differences (biases) between values, the 95% confidence interval limits were fairly wide, particularly for examiner D when compared with the other three examiners (Fig. 5 and Table 3).

**Table 3:** Bias of measurements between different examiners and different methods

	95% Lower Limit	95% Upper Limit	Bias
Examiners			
A to B	−0.912	1.023	0.055
A to C	−1.107	1.099	- 0.004
A to D	−1.559	0.934	−0.313
B to C	- 1.199	1.080	- 0.056
B to D	- 1.656	0.919	- 0.369
C to D	- 1.717	0.718	- 0.309
Methods			
CP to CC	- 1.455	1.060	- 0.198
CP to DP	- 1.244	1.219	- 0.013
CP to DC	- 1.249	1.152	- 0.049
CC to DP	−1.012	1.382	0.185
CC to DC	−0.952	1.250	0.149
DP to DC	−0.652	0.580	−0.036

A, B, C, and D: the four examiners in the study. CP, conventional method using periodontal probe; CC, conventional method of taking measurements on cast model using caliper; DP, digital measurements of intraoral scans; and DC, digital measurements of digitalized cast models

**Fig. 6:**
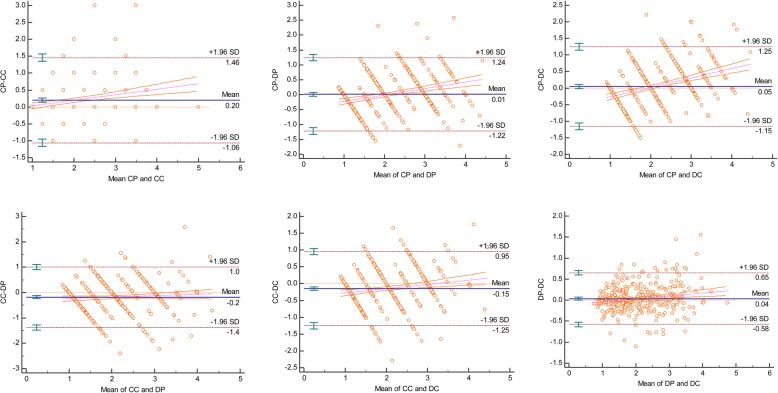
Bland and Altman analysis showing the extent of agreement among the four methods used in this study. CP, conventional method using periodontal probe; CC, conventional method of taking measurements on cast model using caliper; DP, digital measurements of intraoral scans; and DC, digital measurements of digitized cast models

Differences between methods, irrespective of the examiners, are illustrated in the Bland and Altman plots in Fig. 6 and presented in Table 3. The least differences were observed between CP and DP (− 0.013 mm), DP and DC (− 0.036 mm,) followed by CP and DC (− 0.049 mm). Although these differences (biases) were very low, their 95% confidence interval limits were fairly broad, except for the difference between methods DP and DC. The discrepancy was more obvious for CC when compared with the other methods (Fig. 6 and Table 3).

## Discussion

In the present study, the reliability and reproducibility of digital measurements of gingival recession (DP, DC) compared with the conventional methods using dental probe and cast models (CP, CC) were assessed. Digital measurements proved to be more accurate when compared to the clinical measurements with regard to reproducibility between examiners.

In the present study, significant differences were observed in the measurements obtained by CP and CC when compared with those obtained by DP and DC. The highest measurements of gingival recession were achieved using the conventional dental probe method (CP) when compared with the other three methods in the current study. This is in accordance with the findings of the study by Schneider et al. [16], who reported discrepancy in measurements taken by (CP) and attributed this phenomenon to the color difference between the exposed root surface and the enamel, which is more distinctly visible intraorally when compared to the cast or digital models.

Inter-examiner variability was higher between CC and CP methods when compared to methods DP and DC indicating superior reproducibility of measurements when digital methods were used. Similar findings have been reported in previous studies comparing measurements in the oral cavity using conventional and digital methods [16, 17]. Moreover, the lowest inter-examiner agreement was noted with method CP and the highest with method DP. One of the main advantages of using digital technology is that the images can be magnified and viewed from various angles. In addition, the possibility of taking repeated measurements with superior reproducibility will greatly improve the quality of the data collected. In a recent study, the reproducibility of a digital method aimed at evaluating the apico-coronal migration of free gingival margin was validated [18]. Furthermore, the use of a digital model is patient-friendly, as it can reduce both anxiety and discomfort for the patient.

Failure in obtaining accurate measurements of gingival recession can lead to false results and affect the credibility of research studies. Often conventional methods used for these measurements are cost-effective, but have an increased potential for errors due to various factors, such as limited accessibility, manual errors, and variations in the angle of approach. Cast models offer better visual accessibility and the opportunity for repeated measurements. However, additional steps such as impression taking and fabrication of casts may lead to inaccuracies in measurement [16]. Digital measurements have been shown to be more accurate than those obtained using direct oral and cast model methods [16, 17, 19].

Bland and Altman [15] plots aid in calculating the mean of the differences between two measurements. The confidence limits around the mean can be used to assess the extent of variation, which might influence the measurements; mean values closer to zero indicate better agreement between the examiners. On the basis of biases shown in Table 3, it can be implied that the differences in measurement (between examiners and to lesser extent between methods are not clinically significant; the maximum difference did not exceed half a millimeter. However, these differences have somewhat broad 95% confidence intervals extending up to 2 mm which is known to be clinically paramount and may violate the reliability of the used methods. According to McCoy et al. [14], if the ratings of one examiner are consistently higher than the other, the mean will be far from zero, but the confidence interval will be narrow; alternatively, if the disagreement between examiners demonstrates an inconsistent pattern, the mean may be closer to zero but the confidence interval will be wide. In the present study, the means between examiners A and B, A and C, and B and C were closer to zero (0.055, 0004, and 0.056, respectively) with narrow 95% CIs (Table 3 and Fig. 5). As seen in inter-method (Table 2), intra-examiner agreement was nearly similar for examiners A, B, and C (ICC, > 90), while the ICC for examiner D was 0.837 (95% CI, 0.759–0.891). These findings indicate the need for further studies to assess the reliability of measurements among dentists.

Intraoral scanning involves the creation of a 3D image of the structures in the oral cavity using various optical technologies. Computer-aided design/computer-aided manufacturing (CAD/CAM) was introduced by Dr. Francois Duret in 1973 [20]. This system can be used to take direct images from the oral cavity or from models created after impressions are taken from the patients [21]. Recently, this technique was successfully used for volumetric analyses after gingival recession treatment [22]. In another study, Wiranto et al. [19] reported that intraoral scanning is a valid and reliable method with high reproducibility for diagnostic measurements in the field of dentistry. Chalmers et al., [17] reported that the reliability of measurements of dental arch relationships using intraoral 3D scans was superior to that using plaster models. Similar to the findings of their study, the inter-examiner reliability of intraoral scans (DP) was superior to the digital cast models (DC), which in turn were superior to the conventional methods (CP, CC) used in the current study. The present study was not able to identify the method with the most accurate measurements of gingival recession. Nevertheless, the use of digital methods and intraoral scanning for measuring gingival recession has not been explored extensively. The current study corroborates the findings of Schneider et al. [16], and endorses the use of digital technology to assess the outcome of various root coverage procedures.

The limitations for the current study include: First, the lack of repeated measures for the same examiners using the same methods which is essential in assessing intra-examiner reliability. Second, the number of the recession sites was comparatively small. With a larger sample size, more reliable results can be obtained. Finally using a William’s periodontal probe does not allow measurements to be recorded to the tenths of millimeter owing to the fact that it is marked in absolute mm. This can significantly alter the readings of gingival recession as the examiner is obliged to round the measurement to the nearest mm.

## Conclusions

Although mostly minor, there was variability in measurements observed for almost all examiners. Inter-examiner variability was higher for methods CC and CP when compared to DP and DC methods indicating superior reproducibility of measurements using digital technology. Variations between examiners or methods can be reduced considerably using digital methods when compared with the conventional methods. In addition, improved reproducibility of measurements obtained via intraoral scanning will increase the validity of the data and enhance the quality of the study.

## Additional file

Additional file 1: Gingival recession measurments for all methods. (XLSX 19 kb)

## Data Availability

The data sets used and/or analyzed during the current study are included in this published article (and its Additional file 1).

## Abbreviations

3D: Three dimensional
CAD/CAM: Computer-aided design/computer-aided manufacturing
CC: Measurements on cast models using a caliper
CEJ: Cemento-enamel junction
CI: Confidence intervals
CP: Direct measurement of gingival recession using William’s periodontal probe intraorally
DC: Digital measurements on virtual models of dental casts
DP: Digital measurement on virtual models obtained by intraoral scanning
GM: Gingival margin
ICC: Intra-class correlation coefficients
SD: Standard deviations
SE: Standard errors of the measured recessions
