# Correction to: The accuracy and reliability of digital measurements of gingival recession versus conventional methods

**DOI:** 10.1186/s12903-019-0876-4

**Published:** 2019-08-30

**Authors:** Hytham N. Fageeh, Abdullah A. Meshni, Hassan A. Jamal, Reghunathan S. Preethanath, Esam Halboub

**Affiliations:** Division of Periodontics, College of Dentistry, Jazan University, P.O.Box 114, Jazan, 45142 Saudi Arabia

**Correction to: BMC Oral Health (2019) 19: 154**

**https://doi.org/10.1186/s12903-019-0851-0**

After publication of the original article [1], the authors reported that an author’s name has been mispelled. “Helboub” should be replaced with “Halboub”.

The original article has been updated as well.
